# Non‐inferior screen fail rate for persons under the age of 65 in an anti‐amyloid monoclonal antibody clinical trial on Alzheimer’s Disease

**DOI:** 10.1002/alz70859_107109

**Published:** 2025-12-26

**Authors:** Yu‐Jay Huoh, Elly Lee, Dung Trinh, Edward Zamrini, Brenda Martinez, Ralph Lee

**Affiliations:** ^1^ Irvine Clinical Research, Irvine, CA USA; ^2^ Healthy Brain Clinic, Long Beach, CA USA; ^3^ Utah University, Salt Lake City, UT USA

## Abstract

**Background:**

Previous clinical trials for anti‐amyloid monoclonal antibodies in preclinical Alzheimer’s Disease (AD) have reported stopping screening in persons aged 55‐64 (Holdridge, 2024) and/or restricting screening in persons aged 55‐64 to persons with additional risk factors (Sperling, 2020) in favor of participants aged 65+. In this study, we look at the difference in screen failure rates of participants aged 55‐64 relative to participants aged 65+ for a study of early AD within the same class of drug.

**Method:**

In 2024, 185 persons were screened for a Phase 2 clinical trial of an anti‐amyloid monoclonal antibody in early AD across two sites in the Irvine Clinical site network, an independent commercial site network in California. Of these 185 participants, 46 were aged 55‐64, and 139 were aged 65+. The overall screen failure rate was 74.1% (147 screen fails).

**Result:**

For the 46 participants between 55‐64 years old, the screen fail rate was 78.2%. The screen fail rate for participants aged 65+ was 79.9%. A chi‐square test for independence shows that this difference was not statistically significant (x2=‐0.0005, p=0.9828).

Additionally, when running a logistic regression that allows for controlling of demographic variables (years of education, race / ethnicity) and screening location, the effect of being in the 55‐64 age range was actually negative ‐ less likely to screen fail ‐ and also not statistically significant (z = ‐0.642, p = 0.521).

**Conclusion:**

For the early AD study considered in this analysis, participants aged 55‐64 years old did not have an inferior screen fail rate relative to participants aged 65+. Study‐wide decisions to exclude participants aged 55‐64 in preclinical AD may not necessarily be translatable to studies of early AD within the same class of drug and with similar pTau217 screening enrichment strategies.

